# Hypertension is associated with knee osteoarthritis pain in an age-dependent manner☆

**DOI:** 10.1016/j.exger.2025.112938

**Published:** 2025-10-25

**Authors:** Carlos J. Cruz, Javier A. Tamargo, Muhammad Abbas, Samira Capote, Roland Staud, Burel R. Goodin, Roger B. Fillingim, Yenisel Cruz-Almeida

**Affiliations:** a Pain Research and Intervention Center of Excellence, University of Florida, Gainesville, FL, USA; b Department of Community Dentistry and Behavioral Science, College of Dentistry, University of Florida, Gainesville, FL, USA; c Department of Rheumatology and Clinical Immunology, College of Medicine, University of Florida, Gainesville, FL, USA; d Department of Anesthesiology, Washington University School of Medicine, St. Louis, MO, USA

**Keywords:** Hypertension, Osteoarthritis, Pain, Aging

## Abstract

**Objective:**

Osteoarthritis (OA) is a painful and prevalent disease among older adults. Hypertension, commonly comorbid with OA and associated with aging, may influence OA pathology and pain. We hypothesized that hypertension would influence pain outcomes in an age-dependent manner and associate with greater radiographic OA severity among middle-aged and older adults.

**Methods:**

Cross-sectional analysis of 253 adults aged 45–85 years with or without knee pain was performed. Hypertension was defined by self-reported diagnosis or use of antihypertensive medications. Radiographic knee OA was assessed using Kellgren-Lawrence (KL) grades (none/early-stage OA: KL 0–2; late-stage OA: KL 3–4). Pain outcomes included the Western Ontario and McMaster Universities Arthritis Index (WOMAC) and movement-evoked pain following the Short Physical Performance Battery (SPPB).

**Results:**

Females with hypertension had greater odds of late-stage radiographic knee OA (KL grade > 2) than normotensive females (AOR = 3.70, 95 % CI = 1.63–8.37; *p* = 0.002); this was not observed in males. Hypertension was associated with age-dependent decline in WOMAC total scores (β = −1.018, *p* < 0.001) and movement-evoked pain (balance: β = −0.881, *p* = 0.001; walking: β = −1.017, *p* = 0.001), with no change in normotensive individuals. However, hypertensive individuals had greater odds of poor physical performance than normotensive individuals (AOR: 1.96, 95 % CI: 1.08–3.70; *p* = 0.029).

**Conclusions:**

Hypertension was associated with an age-dependent decline in reported knee pain, poorer physical function, and more severe radiographic knee OA in females. These findings suggest hypertensive individuals may deviate from a progressive OA pain trajectory and contribute to discordance between joint damage and pain.

## 1. Introduction

Osteoarthritis (OA) is a painful and debilitating disease that is common among older adults (Zhang and Jordan, 2010). Among these older adults, underlying comorbidities are also more prevalent, which may influence OA pathogenesis. For example, OA is commonly comor- bid with obesity, hypertension, and diabetes (Swain et al., 2020). While the contributions of each to OA pathophysiology are difficult to isolate, hypertension may uniquely play a dual role in both structural joint remodeling and pain sensitivity (Ching et al., 2021; Sacco et al., 2013).

While limited studies have investigated underlying pathophysiologic processes between hypertension and OA, potential drivers have been hypothesized. For example, hypertension may increase intraosseous pressure, lead to joint tissue ischemia, and promote vascular invasion through the osteochondral junction — all of which may contribute to pathologic structural remodeling (Ching et al., 2021; Findlay, 2007). In prior clinical reports, hypertension has been associated with greater radiographic OA severity, with stronger associations noted in females (Lo et al., 2022; Kim et al., 2016). Preclinical reports have also shown that hypertension leads to greater histological evidence of joint damage in OA, also suggesting a greater effect in females (Yeater et al., 2023). Besides structural joint changes, the effect of hypertension on OA pain physiology is not well understood.

Elevated blood pressure can modify pain responses, in what is known as blood-pressure-related hypoalgesia (Zamir and Maixner, 1986). However, the extent of this effect may depend both on pain type (i.e., acute versus chronic) and blood pressure status (i.e., normotensive vs hypertensive). For example, among normotensive individuals, elevated blood pressure is associated with reduced sensitivity to acute pain (Fillingim and Maixner, 1996). In individuals with chronic pain, this effect is thought to be partially diminished (Maixner et al., 1997; de la Coba et al., 2018; Bruehl et al., 2002), although findings are mixed — some have observed a hypoalgesic effect (Hagen et al., 2005), while others have reported a hyperalgesic effect (Liu and Du, 2024). Overall, the extent to which hypertension influences pain in individuals with chronic OA remains poorly understood. Hence, clarifying this relationship could improve our understanding of pain heterogeneity in OA (Kittelson et al., 2014), especially given hypertension’s potential to widen the discordance between reported pain and joint-level pathology (Bedson and Croft, 2008).

Aging is associated with underlying comorbidities (e.g., metabolic syndrome), arterial stiffening, and hormonal shifts, all of which are linked to hypertension (Sun, 2015). Therefore, the relationship between pain and blood pressure may be age-dependent. Since prior work examining the relationship between chronic pain and hypertension is conflicting (i.e., hypoalgesia vs hyperalgesia) (Hagen et al., 2005; Liu and Du, 2024), in the present study we aimed to consider the influence of age in this relationship. Specifically, we hypothesized that hypertension would influence pain responses in an age-dependent manner and be associated with greater radiographic evidence of OA.

## 2. Materials and methods

### 2.1. Study design

This study involved a cross-sectional analysis of a multisite study evaluating community-dwelling middle-aged and older individuals (45–85 years) with and without symptomatic knee pain who were recruited using community- and clinic-based methods from areas surrounding the University of Florida (UF; Gainesville, FL) and the University of Alabama at Birmingham (UAB; Birmingham, AL). All procedures were approved by the Institutional Review Boards at both sites, with the UF serving as the IRB of record (UF IRB01); participants provided written informed consent to participate and were compensated for participation. The participants (*N* = 253) included non-Hispanic Black and non-Hispanic White males and females; the sample size varied slightly for each reported outcome (Table 1) due to occasional missing data (e.g., incomplete radiographs or pain ratings). Participant eligibility was determined through an initial phone screening, followed by an in-person baseline health assessment session. Exclusion criteria included: 1) uncontrolled hypertension (blood pressure > 150/95 mmHg measured during the in-person visit prior to enrollment); 2) significant surgical operations to the index (i.e., most painful) knee; 3) systemic rheumatic disease (e.g., rheumatoid arthritis, gout, systemic lupus erythematosus, and fibromyalgia); 4) neurological disease (e.g., Parkinson's disease, multiple sclerosis); 5) chronic opioid use; 6) peripheral neuropathy; 7) pregnant or nursing; 8) had more severe pain in body sites other than the index knee; 9) hospitalization for psychiatric disorders in the preceding year; and 10) heart disease (e.g., congestive heart failure, acute myocardial infarction).

### 2.2. Hypertension and diabetes status

Participants were categorized as having hypertension if they reported a current diagnosis of hypertension or were taking one or more antihypertensive medications; those who met neither criterion were considered not to have hypertension. Diabetic status was defined similarly. Additionally, arterial blood pressure (including systolic, diastolic, and mean arterial pressure) was measured three times during the inperson session, and the average of these readings is reported in Table 1.

### 2.3. Radiographic knee OA severity as assessed by KL grade

Posterior-anterior and lateral knee radiographs of the index (most painful) knee were obtained during the in-person health assessment session. Radiographs from both study sites were graded for knee OA severity by a rheumatologist (RS) who was blinded to the study participants using the Kellgren-Lawrence (KL) classification system (Kellgren and Lawrence, 1957). Radiographs were assigned the following grades: 0 = normal; 1 = doubtful; 2 = mild; 3 = moderate; 4 = severe. To evaluate whether hypertensive status was associated with OA severity at the opposite ends of the grading scale, KL grades were dichotomized into none/early-stage OA (grades 0–2) and late-stage OA (grades 3–4).

### 2.4. Clinical pain: Western Ontario and McMaster Universities Osteoarthritis Index (WOMAC)

Participants completed the WOMAC questionnaire to assess joint pain, stiffness, and physical function (Bellamy et al., 1988). The subscale for pain ranges of 0–20, 0–8 for stiffness, and 0–68 for physical function. Scores from each subscale were summed to calculate the total WOMAC score.

### 2.5. Short Physical Performance Battery (SPPB) assessment and pain ratings

The Short Physical Performance Battery (SPPB) was administered to participants during the in-person health assessment session by trained study staff. The SPPB consisted of three components: timed balance tasks, a timed walking task performed at a normal pace, and a series of chair-stand tasks (Guralnik et al., 1995). Total scores ranged from 0 to 12, with higher scores indicating better physical performance. SPPB scores were dichotomized into two categories: 0–9 and 10–12. These cutoffs were selected as an SPPB score of less than 10 (or ≤9) is predictive of mobility disability (Pavasini et al., 2016). To assess movement-evoked pain, participants rated their pain on a numerical rating scale from 0 to 100 (0 = no pain; 100 = most intense pain imaginable) after completing each component of the battery.

### 2.6. Statistical analysis

Binary logistic regression analyses were conducted using SPSS (v. 30.0.0.0). Linear models, marginal estimates, and all visualizations were performed and generated using RStudio (v. 4.0.2). All models were adjusted for the following covariates: BMI (per 5-unit increase), site (i.e., study location), race, age (per 1-unit increase), sex, diabetes status, and radiographic knee OA as assessed by KL grade. KL grade was included as a covariate in all models except when it served as the outcome variable. We used complete-case (listwise) analysis (no imputation), so sample sizes varied by measure due to occasional missing data (e.g., incomplete radiographs or pain ratings). As a result, the total number of participants included in each model (of N = 253) was as follows: KL grade (n = 247), WOMAC total score (n = 245), SPPB walking pain (n = 245), SPPB balance pain (n = 243), SPPB chair-stand pain (n = 236), and SPPB score (n = 247).

Univariate analysis examining the effect of each covariate on outcome measures is provided in the Supplemental Materials: KL grade (Table S1), WOMAC Total score (Table S2), movement-evoked pain following SPPB tasks (Table S3), and SPPB performance category (Table S4).

## 3. Results

### 3.1. Study demographics

Participants (N = 242–253) were stratified into two groups: normotensive and hypertensive. Overall, individuals with hypertension were older (59.2 ± 8.2 years, mean ± SD) and had a higher BMI (32.9 ± 7.7) than normotensive individuals (56.8 ± 7.8 years and 30.2 ± 7.3; *p* = 0.029 and *p* = 0.004, respectively). Individuals with hypertension also had significantly greater blood pressure compared to normotensive individuals, including systolic (130.0 ± 17.0 vs. 122.8 ± 15.5 mmHg, *p* < 0.001), diastolic (76.6 ± 10.0 vs. 74.0 ± 10.4 mmHg, *p* = 0.042), and mean arterial pressure (93.1 ± 9.9 vs. 88.3 ± 10.5 mmHg, *p* < 0.001). Race was significantly different among individuals with and without hypertension (*p* < 0.001). Specifically, most normotensive individuals were non-Hispanic White (67.9 %), while non-Hispanic Black individuals were more evenly represented in normotensive (45.9 %) and hypertensive (54.1 %) groups. Diabetes status was significantly different (*p* < 0.001), with most hypertensive individuals being diabetic (78.1 %) and most normotensive individuals being non-diabetic (62.4 %). No significant differences were observed in the representation of sex (*p* = 0.221) or study site (*p* = 0.153) between hypertensive and normotensive individuals. Radiographic knee OA severity (KL grade) and symptom measures are summarized in Table 1 and described in detail in subsequent results sections.

### 3.2. Hypertension is associated with late-stage radiographic OA in females but not males

Hypertension was associated with greater odds of late-stage radiographic knee OA (KL grade > 2) compared to normotensive individuals (unadjusted OR = 2.88, 95 % CI = 1.63–5.10, *p* < 0.001). This association remained significant after adjusting for covariates (AOR = 1.96, 95 % CI = 1.03–3.74, *p* = 0.040).

Additionally, the association of hypertension with radiographic knee OA was moderated by sex (interaction, *p* = 0.009). Specifically, hypertensive females had greater odds of late-stage radiographic knee OA (KL grade > 2) compared to normotensive females (AOR = 3.70, 95 % CI = 1.63–8.37; *p* = 0.002), whereas no association was observed in males (AOR = 0.63, 95 % CI = 0.22–1.83; *p* = 0.398, Table 2).

### 3.3. WOMAC total score declined with age in hypertensive individuals

**Fig. 1.**
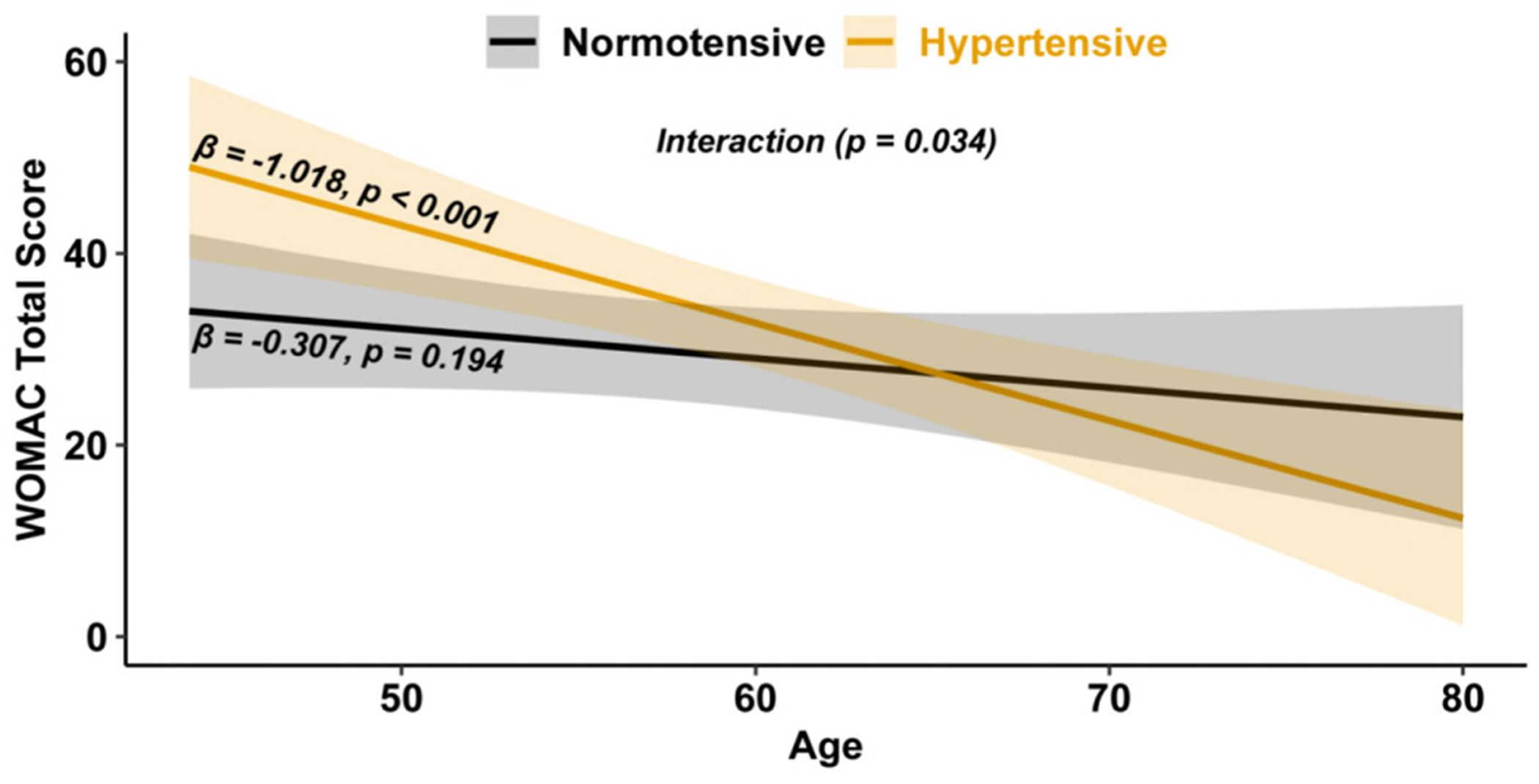
WOMAC total scores across age by hypertension status (N = 245). Marginal estimates are adjusted for site, race, sex, BMI (per 5-unit increase), age (per 1-unit increase), diabetes, KL grade, and hypertension-by-age interaction. Lines represent predicted means; shaded bands indicate 95 % confidence intervals. Statistical significance is denoted by *p* < 0.05; β values = slopes.

Group means from univariate regression analysis revealed higher WOMAC total scores in hypertensive (33.4 ± 4.3, mean ± 95 % CI) compared to normotensive individuals (23.5 ± 3.7; *p* < 0.001). After adjusting for covariates, this difference was no longer significant (hypertensive: 34.3 ± 4.7 vs. normotensive: 29.3 ± 5.2; *p* = 0.091). However, age significantly moderated the relationship between hypertension and WOMAC total score (interaction, *p* = 0.034; Fig. 1). Specifically, hypertensive individuals showed a significant decrease in WOMAC total scores with increasing age (β = −1.018, *p* < 0.001). In contrast, scores remained stable across age in normotensive individuals (β = −0.307, *p* = 0.194, Fig. 1).

### 3.4. Movement-evoked pain declined with age in hypertensive individuals

Group means from unadjusted regression analysis revealed that physical performance pain rating were higher in hypertensive (chair stand: 24.0 ± 5.2; walking: 20.3 ± 4.8, mean ± 95 %CI) than normotensive individuals (chair stand: 16.9 ± 4.3, *p* = 0.040; walking: 13.7 ± 4.2, *p* = 0.046); pain ratings following balance task were not significantly different (hypertensive: 17.8 ± 4.3; normotensive: 12.4 ± 3.8; *p* = 0.064). After adjusting for covariates, physical performance pain ratings did not significantly differ between hypertensive (chair stand: 26.1 ± 6.1; walking: 20.1 ± 5.5; balance: 18.8 ± 4.9) and normotensive (chair stand: 24.3 ± 6.5, *p* = 0.607; walking: 18.0 ± 6.1, *p* = 0.541; balance: 17.3 ± 5.4, *p* = 0.618) individuals.

**Fig. 2.**
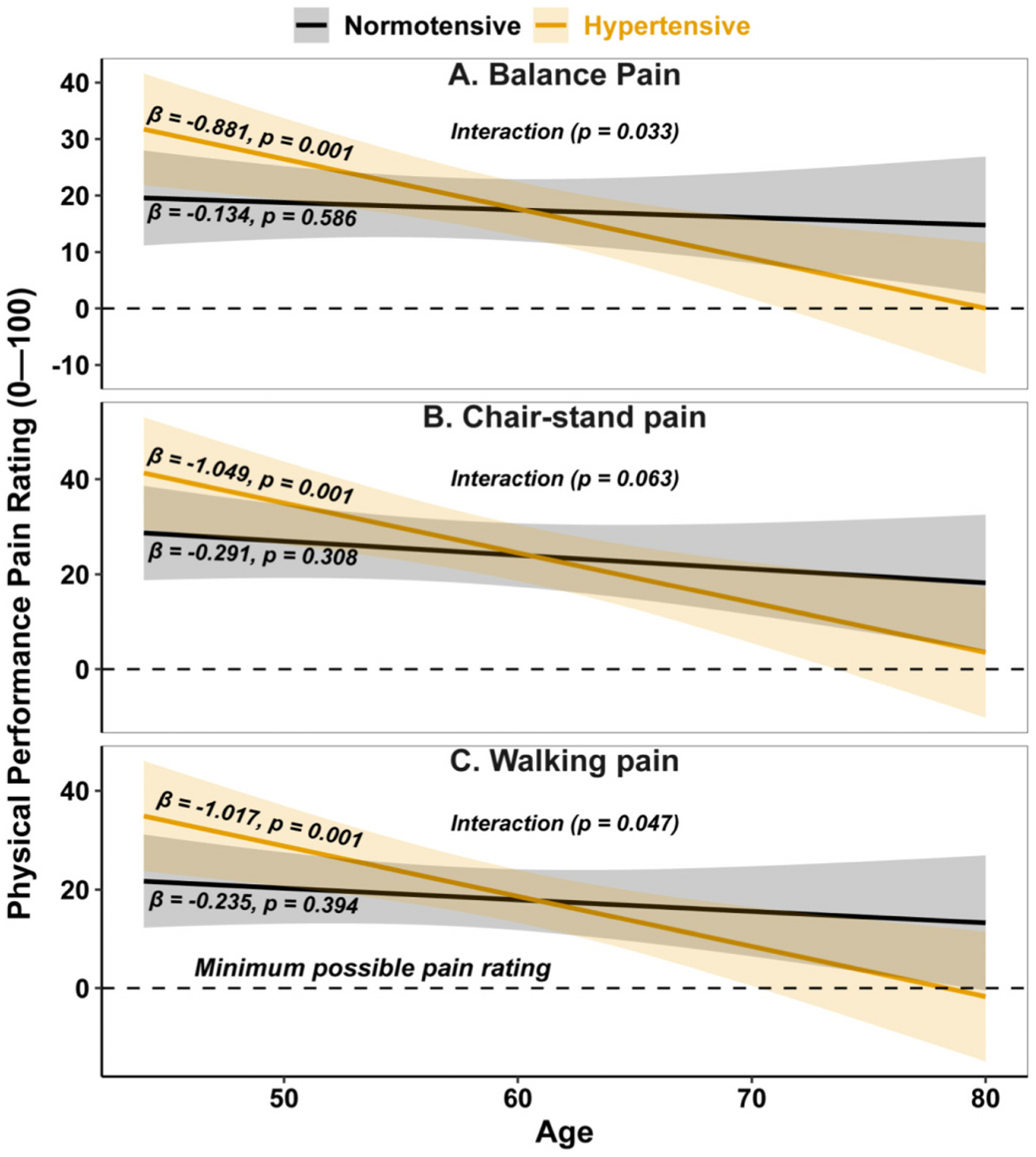
Movement-evoked pain ratings for balance, chair stand, and walking tasks across age, by hypertensive status. Marginal estimates are adjusted for site, race, sex, BMI (per 5-unit increase), age (per 1-unit increase), diabetes, KL grade, and hypertension-by-age interaction. Lines represent predicted means; shaded bands indicate 95 % confidence intervals. The dashed line marks the minimum possible pain rating (0 of 100). Statistical significance is denoted by p < 0.05; β values = slopes.

However, age significantly moderated the relationship between hypertension and pain ratings following balance (interaction, *p* = 0.033, Fig. 2A) and walking (interaction, *p* = 0.047, Fig. 2C) tasks. Specifically, hypertensive individuals reported decreasing pain ratings with increasing age (balance: β = −0.881, *p* = 0.001; walking: β = −1.017, *p* = 0.001), whereas pain ratings remained stable with age in normotensive individuals (balance: β = −0.134, *p* = 0.586, Fig. 2A; walking: β = −0.235, *p* = 0.394; Fig. 2B). The interaction between age and hypertensive status on chair-stand-related pain ratings showed a similar pattern but did not reach statistical significance (*p* = 0.063, Fig. 2B).

### 3.5. Hypertensive individuals had poorer physical performance

Hypertensive individuals had significantly greater odds of poorer physical performance (SPPB score ≤ 9) compared to normotensive individuals (unadjusted OR: 2.27, 95 %CI: 1.36–3.79; *p* = 0.002). This effect remained after adjusting for covariates (AOR: 2.00, 95 % CI: 1.07–3.60; *p* = 0.029). Age did not moderate this relationship (interaction, *p* = 0.829).

## 4. Discussion

Hypertension is strongly associated with OA pathogenesis, with prior reports suggesting a stronger association in females (Ching et al., 2021; Findlay, 2007; Lo et al., 2022; Yeater et al., 2023). Beyond joint-level remodeling, hypertension may influence pain physiology (Sacco et al., 2013; Zamir and Maixner, 1986; de la Coba et al., 2018; Hagen et al., 2005; Liu and Du, 2024). However, the overall direction of hypertension's effect on chronic OA pain is not well understood, with some reports suggesting a hypoalgesic effect (Hagen et al., 2005) and others reporting a hyperalgesic effect (Liu and Du, 2024). Given that aging influences blood pressure regulation, age is likely to influence the relationship between hypertension and pain (Sun, 2015). To address this gap, we conducted a cross-sectional analysis of middle-aged and older adults with and without knee pain to evaluate whether hypertension was associated with OA-related pain and radiographic severity, and whether these relationships were moderated by age or sex. Our findings suggest that hypertension is associated with OA-related pain in an agedependent manner; that is, hypertensive individuals reported lower pain ratings with increasing age. We also found that hypertension was associated with radiographic OA severity in a sex-dependent manner, with hypertension being associated with late-stage OA only among females.

Hypertension has been associated with greater radiographic knee OA, with a stronger effect in females (Lo et al., 2022). Here, we also observe a similar effect, where hypertensive females, but not males, showed greater odds of having greater radiographic knee OA (KL grade > 2) compared to their normotensive counterparts. While the underlying pathophysiologic drivers were not explored in our analysis, sex hormones have been associated with protective effects on joint health (Gulati et al., 2023; Fernandes and Valdes, 2015) and blood pressure control (Prabhushankar et al., 2014; Rossi et al., 2019). Hormonal changes in older women (e.g., postmenopausal decline in estrogen) may influence shared molecular pathways between the renin-angiotensin- aldosterone system and bone and cartilage remodeling (Ching et al., 2021; Nwia et al., 2023), potentially explaining the sex-dependent results observed. Ultimately, future mechanistic studies are needed to characterize the sex-dependent drivers of comorbid OA and hypertension. Overall, our results highlight the need for early intervention strategies to slow OA progression in females with hypertension, who may be at greater risk for severe knee OA and ultimately joint failure.

Prior work exploring the effect of hypertension on chronic pain has reported both hypoalgesic (Hagen et al., 2005) and hyperalgesic (Liu and Du, 2024) effects. Our findings indicate that this relationship is moderated by age. Middle-aged individuals with hypertension reported greater joint pain (WOMAC total), while older-aged individuals with hypertension reported less pain. In contrast, normotensive individuals showed stable pain ratings across ages. Overall, these results suggest that hypertension may be associated with hypoalgesia or hyperalgesia, depending on age. Co-occurring physiologic processes, such as arterial stiffness (Sun, 2015) and hormonal shifts (Gulati et al., 2023; Colafella and Denton, 2018), may partially explain these patterns, as the underlying drivers of hypertension in middle-aged versus older adults may differentially influence pain processing. Although our model accounted for demographic and metabolic covariates (race, sex, diabetes, and BMI), other unmeasured lifestyle (e.g., nutrition) (Bazzano et al., 2013; Elma et al., 2022) and psychosocial (e.g., chronic stress) (Cuevas et al., 2017; Edwards et al., 2016) factors may influence hypertension and pain and therefore contribute to these age-dependent pain differences. Future studies may also consider the duration of hypertension, which may be an important modifier. For example, prolonged hypertension in the context of age-related arterial stiffening may shift baroreflex set point (i.e., reduce baroreflex sensitivity) and interact with central pain-modulatory circuits, potentially altering cardiovascular-pain coupling over time and ultimately pain sensitivity (Zamir and Maixner, 1986; Suarez-Roca et al., 2021; Guasti et al., 2002; Ottaviani et al., 2018). Ultimately, large longitudinal epidemiologic studies that track within-person change over an extended period (i.e., decades) are needed to identify whether hypertensive status and its chronicity predict distinct OA pain trajectories. In that context, previously estimated minimal clinically important differences (MCIDs) for WOMAC total (approximately 16–17 points) can serve as benchmarks (Kim et al., 2021; Taslakian et al., 2023). Overall, these results suggest that hypertension may exert a hyperalgesic effect in middle-aged adults and a hypoalgesic effect in older-aged adults.

Hypertension’s role in pain is thought to differ in acute versus chronic pain (Sacco et al., 2013; de la Coba et al., 2018; Bruehl et al., 2002). In our analysis, movement-evoked pain (MEP) ratings followed a similar age-dependent pattern as captured using WOMAC total score. Specifically, among hypertensive individuals, middle-aged adults reported higher MEP, while older individuals reported lower MEP. In normotensive individuals, MEP ratings remained stable across age. Interestingly, despite this age-related decline in pain ratings, individuals with hypertension still demonstrated poorer physical performance (i.e., SPPB ≤ 9), which is predictive of mobility disability (Pavasini et al., 2016). Although prior studies suggest that greater movement-evoked pain is associated with poorer physical performance scores (Booker et al., 2019; Cruz-Almeida et al., 2017), this relationship may not hold in a hypertensive phenotype. Future studies incorporating real-time assessments (e.g., wearable devices that capture physical activity, selfreported pain, and cardiovascular parameters) could help clarify the potential dissociation between pain reporting and physical performance in individuals with hypertension. Furthermore, future longitudinal epidemiologic analyses could clarify whether within-person changes in MEP over decade-scale periods, particularly among individuals with chronic hypertension, approach estimated MCID thresholds (e.g., a 1.1-point decrease out of 10 in pain rating) (Fleagle et al., 2024).

Several limitations should be considered when interpreting our results. First, our analysis was cross-sectional and cannot establish temporality or causality between hypertension and the reported pain or radiographic OA outcomes. Radiographic KL grades were dichotomized (KL 0–2 vs KL 3–4) to focus on late-stage radiographic OA (KL > 2) and to preserve power, which reduces grade-specific resolution. Longitudinal analyses are needed to assess whether changes in hypertensive status predict the age-related pain relationship observed in this study and to evaluate whether hypertension is associated with accelerated radiographic OA progression within specific KL grades (e.g., transition from KL 2 to 3). While the measures for our primary outcomes (WOMAC, SPPB, and MEP) are well-validated to assess OA symptoms and lower-extremity physical function, they are limited in their ability to capture chronicity or variations over time. Additionally, our analysis is retrospective, and uncontrolled hypertension was an exclusion criterion for recruitment into the original study. While this limits our ability to assess the sensitivity of large blood pressure variations in our analyses, it also focuses our observations to a clinically relevant group — that is, individuals with diagnosed and/or medically managed hypertension. Furthermore, while our models adjusted for factors associated with higher hypertension prevalence, such as greater BMI, diabetes, and race (Table 1), other comorbidities (e.g., dyslipidemia) and social demographics may also impact the findings (Pan et al., 2020; Alenazi et al., 2020; Mickle et al., 2020; Bartley et al., 2019). Ultimately, the observations reported herein are limited to a single, cross-sectional observation in individuals with or without controlled hypertension.

Overall, our findings suggest that age moderates the association between hypertension and knee pain, with hypertensive individuals reporting less pain at older ages. In contrast, knee pain levels remained stable with age in normotensive individuals. Additionally, hypertension was associated with poorer physical performance overall and with more severe radiographic OA in hypertensive females, but not males. Our findings suggest that clinical risk stratification, particularly in postmenopausal women with hypertension, may facilitate earlier identification and management of OA, as symptoms may not fully reflect radiographic severity in this group. Future work should consider how hypertension may contribute to the discordance between joint pathology and pain in OA (Bedson and Croft, 2008), and whether hypertension-related treatment can influence these relationships. Ultimately, our findings support that both hypertensive status and age contribute to the heterogeneity of knee pain and should be considered when phenotyping individuals with OA.

## Supplementary Material

Supplementary Tables

## Figures and Tables

**Table 1:** Participant characteristics.

		Overall (N = 253) No (%) Mean ± SD	Normotensive (N = 145) No (%) Mean ± SD	Hypertensive (N = 108) No (%) Mean ± SD	*p*-Value
***Demographics***					
Age (years)		57.8 ± 8.0	56.8 ± 7.8	59.2 ± 8.2	0.019^3^
BMI (kg/m^2^)		31.4 ± 7.6	30.2 ± 7.3	32.9 ± 7.7	0.004^3^
Systolic blood pressure (mmHg)		125.9 ± 16.5	122.8 ± 15.5	130.0 ± 17.0	<0.001^3^
Diastolic blood pressure (mmHg)		75.1 ± 10.3	74.0 ± 10.4	76.6 ± 10.0	0.042^3^
Mean arterial blood pressure (mmHg)		90.4 ± 10.5	88.3 ± 10.5	93.1 ± 9.9	<0.001^3^
Sex	Male	92 (38.7)	48 (52.2)	44 (47.8)	0.211^4^
	Females	161 (63.6)	97 (60.2)	64 (39.8)	
Site	University of Florida (UF)	158 (62.5)	96 (60.8)	62 (39.2)	0.153^4^
	University of Alabama at Birmingham (UAB)	95 (37.5)	49 (51.6)	46 (48.4)	
Race	Non-Hispanic Black	122 (48.2)	56 (45.9)	66 (54.1)	<0.001^4^
	Non-Hispanic White	131 (51.8)	89 (67.9)	42 (32.1)	
Diabetes status	Nondiabetic	221 (87.4)	138 (62.4)	83 (37.6)	<0.001^4^
	Diabetic	32 (12.6)	7 (21.9)	25 (78.1)	
***Radiographic knee OA severity***					
KL grade category	0–2 (early OA)	177 (71.7)	114 (64.4)	63 (35.6)	<0.001^4^
	3–4 (late OA)	70 (28.3)	27 (38.6)	43 (61.4)	
***Symptoms***					
SPPB score	0–9 (<10)	146 (57.7)	96 (65.8)	50 (34.2)	0.002^4^
	10–12	107 (42.3)	49 (45.8)	58 (54.2)	
WOMAC total score^1^		27.7 ± 23.1	23.5 ± 21.6	33.4 ± 23.9	<0.001^3^
SPPB walking MEP^2^		16.6 ± 25.8	13.8 ± 23.2	20.3 ± 28.5	0.053^3^
SPPB balance MEP^2^		14.7 ± 22.8	12.4 ± 20.0	17.8 ± 25.9	0.074^3^
SPPB chair stand MEP^2^		19.8 ± 26.6	16.9 ± 23.7	24.0 ± 29.9	0.048^3^

MEP = movement-evoked pain.

1 n = 251.

2 Walking: n = 251, balance: n = 247, chair stand: n = 242.

3 *t*-Test.

4 Chi squared test.

**Table 2:** Adjusted odds of late-stage radiographic knee OA (KL 3–4 vs KL 0–2) in hypertensive versus normotensive males and females.

Predictor	Strata^1^	AOR (95 % CI)^2^	*p*-Value
Hypertension-by-sex interaction	Hypertensive female	3.70 (1.63–8.37)	0.002
	Hypertensive male	0.63 (0.22–1.83)	0.398

1 Reference category = normotensive males or normotensive females.

2 Binary logistic regression model adjusted for BMI, site, race, age, sex, and diabetes status, with hypertension, sex, and hypertension-by-sex interaction in the model.

## Data Availability

Data will be made available on request.

## Appendix A. Supplementary data

Supplementary data to this article can be found online at https://doi.org/10.1016/j.exger.2025.112938.
