# Supplementary material for: Hypertension is associated with knee osteoarthritis pain in an age-dependent manner
Source: Exp Gerontol. Author manuscript; Available in PMC 2026 May 15. (PMC13176664; doi:10.1016/j.exger.2025.112938)
Supplement: Supplementary Tables [file NIHMS2167799-supplement-Supplementary_Tables.docx]

**Supplementary Material**

| Table S1. Univariate Binary Logistic Regression Analysis of Radiographic Knee OA (KL 3–4 vs KL 0–2) | | |
| --- | --- | --- |
| Predictor | unadjusted OR (95%CI) | p-value |
| age (per 1-unit increase) | 1.04  (1.00 – 1.07) | 0.040 |
| site (UAB, *reference: UF*) | 1.89  (1.08 – 3.32) | 0.027 |
| race (NHW, *reference: NHB*) | 0.62  (0.35 – 1.08) | 0.092 |
| sex (female, *reference: male*) | 1.25  (0.69 – 2.23) | 0.463 |
| BMI (per 5-unit increase) | 1.58  (1.29 – 1.94) | < 0.001 |
| Diabetes (diabetic, *reference: nondiabetic*) | 1.90  (0.88 – 4.09) | 0.102 |
| ^1^ KL grade categorized as none/early-stage radiographic knee OA (KL 0–2) and late-stage radiographic knee OA (KL 3–4).  ^2^ OR > 1 indicates increased odds of late-stage radiographic knee OA. | | |

| Table S2. Univariate Regression Analysis of WOMAC Total Score | | |
| --- | --- | --- |
| Predictor | β (95% CI) | p-value |
| age (per 1-unit increase) | -0.447  (-0.830 to -0.124) | 0.008 |
| site (UAB, *reference: UF*) | 3.055  (-2.879 to 8.989) | 0.312 |
| race (NHW, *reference: NHB*) | -14.709  (-20.167 to -9.249) | < 0.001 |
| sex (female, *reference: male*) | 0.946  (-5.025 to 6.917) | 0.755 |
| BMI (per 5-unit increase) | 4.431  (2.608 to 6.253) | < 0.001 |
| Diabetes (diabetic, *reference: nondiabetic*) | 11.027  (2.509 to 19.546) | 0.011 |
| KL Grade  (per 1-grade increase) | 18.028  (11.684 to 24.372) | < 0.001 |
| ^1^ β > 0 indicates greater WOMAC total score compared to reference group. | | |

| Table S3. Univariate Regression Analysis of Movement-Evoked Pain Ratings | | | | | |  |
| --- | --- | --- | --- | --- | --- | --- |
| Predictor | Balance Pain | | Chair-Stand Pain | | Walking Pain | |
|  | β (95% CI) | p-value | β (95% CI) | p-value | β  (95% CI) | p-value |
| age (per 1-unit increase) | -0.400  (-0.751 to -0.050) | 0.026 | -0.424  (-0.836 to -0.013) | 0.043 | -0.528  (-0.921 to -0.135) | 0.009 |
| site (UAB, *reference: UF*) | 7.587  (1.755 to 13.420) | 0.011 | 7.370  (0.446 to 14.293) | 0.037 | 10.610  (4.113 to 17.107) | 0.002 |
| race (NHW, *reference: NHB*) | -12.848  (-18.325 to -7.372) | < 0.001 | -15.151  (-21.639 to -8.662) | < 0.001 | -14.117  (-20.295 to -7.939) | < 0.001 |
| sex (female, *reference: male*) | -0.007  (-5.944 to 5.930) | 0.998 | 3.349  (-3.641 to 10.339) | 0.346 | 3.905  (-2.753 to 10.562) | 0.249 |
| BMI (per 5-unit increase) | 2.856  (0.996 to 4.715) | 0.003 | 2.988  (0.717 to 5.081) | 0.009 | 2.678  (0.589 to 4.768) | 0.012 |
| Diabetes (diabetic, *reference: nondiabetic*) | 7.808  (-0.659 to 16.274) | 0.071 | 9.761  (-1.073 to 20.594) | 0.077 | 2.324  (-7.293 to 11.942) | 0.632 |
| KL Grade  (per 1-grade increase) | 14.211  (7.821 to 20.601) | < 0.001 | 20.839  (13.264 to 28.414) | < 0.001 | 18.533  (11.335 to 25.731) | < 0.001 |
| ^1^ β > 0 indicates greater pain rating compared to reference group. | | | | | | |

| Table S4. Univariate Binary Logistic Regression Analysis of Short Physical Performance Battery Category | | |
| --- | --- | --- |
| Predictor | unadjusted | p-value |
|  | OR (95%CI) |  |
| age (per 1-unit increase) | 0.99 (0.95 – 1.03) | 0.562 |
| site (UAB, *reference: UF*) | 0.35 (0.18 – 0.65) | <0.001 |
| race (NHW, *reference: NHB*) | 0.54 (0.30 – 0.97) | 0.038 |
| sex (female, *reference: male*) | 1.25 (0.69 – 2.27) | 0.454 |
| BMI (per 5-unit increase) | 1.35 (1.08 – 1.69) | 0.009 |
| Diabetes (diabetic, *reference: nondiabetic*) | 1.76 (0.74 – 4.22) | 0.203 |
| KL Grade (per 1-grade increase) | 1.20 (1.01 – 1.44) | 0.040 |
| ^1^ SPPB ≤ 9 classified as low physical performance.  ^2^ OR > 1 indicates increased odds of poor physical performance. | | |
